# Primary tumor regression speed after radiotherapy and its prognostic significance in nasopharyngeal carcinoma: a retrospective study

**DOI:** 10.1186/1471-2407-14-136

**Published:** 2014-02-27

**Authors:** Ning Zhang, Shao-Bo Liang, Yan-Ming Deng, Rui-Liang Lu, Hai-Yang Chen, Hai Zhao, Zhi-Qian Lv, Shao-Qiang Liang, Lin Yang, Dong-Sheng Liu, Yong Chen

**Affiliations:** 1 Radiotherapy Department of Nasopharyngeal Carcinoma, Cancer Center, The First People’s Hospital of Foshan, 81 Lingnan Street North, Foshan, People’s Republic of China; 2 Department of Radiation Oncology, State Key Laboratory of Oncology in South China, Sun Yat-sen University Cancer Center, 651 Dongfeng Road East, Guangzhou 510060, People’s Republic of China; 3 Chemotherapy Department of Head & Neck & Chest Carcinoma, Cancer Center, The First People’s Hospital of Foshan, 81 Lingnan Street North, Foshan, People’s Republic of China; 4 Department of Imaging Diagnosis, The First People’s Hospital of Foshan, 81 Lingnan Street North, Foshan, People’s Republic of China; 5 Department of Medical Statistics, The First People’s Hospital of Foshan, 81 Lingnan Street North, Foshan, People’s Republic of China

**Keywords:** Nasopharyngeal carcinoma, Radiotherapy, Tumor regression, Survival

## Abstract

**Background:**

To observe the primary tumor (PT) regression speed after radiotherapy (RT) in nasopharyngeal carcinoma (NPC) and evaluate its prognostic significance.

**Methods:**

One hundred and eighty-eight consecutive newly diagnosed NPC patients were reviewed retrospectively. All patients underwent magnetic resonance imaging and fiberscope examination of the nasopharynx before RT, during RT when the accumulated dose was 46–50 Gy, at the end of RT, and 3–4 months after RT.

**Results:**

Of 188 patients, 40.4% had complete response of PT (CRPT), 44.7% had partial response of PT (PRPT), and 14.9% had stable disease of PT (SDPT) at the end of RT. The 5-year overall survival (OS) rates for patients with CRPT, PRPT, and SDPT at the end of RT were 84.0%, 70.7%, and 44.3%, respectively (*P* < 0.001, hazard ratio [HR] = 2.177, 95% confidence interval [CI] = 1.480-3.202). The 5-year failure-free survival (FFS) and distant metastasis-free survival (DMFS) rates also differed significantly (87.8% vs. 74.3% vs. 52.7%, *P* = 0.001, HR = 2.148, 95% CI, 1.384-3.333; 91.7% vs. 84.7% vs. 66.1%, *P* = 0.004, HR = 2.252, 95% CI = 1.296-3.912). The 5-year local relapse–free survival (LRFS) rates were not significantly different (95.8% vs. 86.0% vs. 81.8%, *P =* 0.137, HR = 1.975, 95% CI, 0.976-3.995). By multivariate analyses, the PT regression speed at the end of RT was the only independent prognostic factor of OS, FFS, and DMFS (*P <* 0.001, *P =* 0.001, and *P =* 0.004, respectively). The 5-year FFS rates for patients with CRPT during RT and CRPT only at the end of RT were 80.2% and 97.1%, respectively (*P =* 0.033). For patients with persistent PT at the end of RT, the 5-year LRFS rates of patients without and with boost irradiation were 87.1% and 84.6%, respectively (*P =* 0.812).

**Conclusions:**

PT regression speed at the end of RT was an independent prognostic factor of OS, FFS, and DMFS in NPC patients. Immediate strengthening treatment may be provided to patients with poor tumor regression at the end of RT.

## Background

Radiotherapy (RT) is the first choice and main treatment for newly diagnosed and nonmetastatic nasopharyngeal carcinoma (NPC). Intensity-modulated RT (IMRT), an important milestone in RT development, can not only increase local control, but also reduce RT-related toxicities in NPC [1-3]. Distant metastasis is the main reason for treatment failure [4]. Identifying the high-risk group for distant metastasis and increasing chemotherapy intensity are the most important approaches to increasing the overall survival (OS) rate in NPC patients.

The main prognostic factors of NPC widely used in clinical work include the staging system, tumor size, and plasma Epstein-Barr virus (EBV) DNA level [5-12]. Currently, the 7^th^ edition of the American Joint Committee on Cancer (AJCC) staging system is used to predict prognoses and determine treatment strategies worldwide [5]. Using the 7^th^ edition system instead of the 6^th^ edition system, Chen et al. observed better segregation of survival curves in NPC patients [6]. Previous research has demonstrated that tumor sizes, including primary tumor (PT) volume and maximum PT diameter, could serve as important prognostic factors in NPC [7-9]. Chong et al. indicated that it might be possible to incorporate tumor volume as an additional prognostic factor within the existing TNM staging system [10]. The plasma EBV DNA level is useful for predicting prognosis and evaluating treatment failure in NPC [11]. In patients with Stage I–II NPC, pretherapy plasma EBV DNA levels identified a high-risk group with a probability of distant failure similar to that of patients with advanced-stage disease [12].

All of these pretreatment factors, which reflect severity of disease and predict prognosis, form the basis of determining treatment strategies. The Response Evaluation Criteria in Solid Tumors (RECIST) is used to evaluate the response of tumor regression. Overall responses are divided into four levels: complete response (CR), partial response (PR), stable disease (SD), and progressive disease (PD) [13]. The relationship between recent tumor regression and long-term survival in NPC patients remains unknown. If poor tumor regression can predict potential treatment failure, such patients may require timely strengthening treatment.

Therefore, we performed a retrospective study to observe PT regression speed after RT and to evaluate its prognostic significance in NPC patients. The purpose of this study was to investigate whether patients with poor tumor regression require strengthening treatment, thereby increasing the effectiveness of treatment. Our data may improve understanding of the biological nature of NPC.

## Methods

### Study population

This retrospective study was approved by the ethics committee of the First People’s Hospital of Foshan, Foshan, China. The inclusion criteria were as follows: patients with newly diagnosed, nonmetastatic, and histologically proven NPC. Patients were treated with RT alone or concurrent chemoradiotherapy (CCRT), but did not receive neoadjuvant chemotherapy or adjuvant chemotherapy. In addition, all patients underwent nasopharyngeal fiberscope and magnetic resonance imaging (MRI) scan of the nasopharynx before RT, during RT when the accumulated dose was 46–50 Gy, at the end of RT, and 3–4 months after RT. Between June 2004 and November 2009, 188 eligible patients were included in the study, including 138 male and 50 female patients (male:female, 2.8:1). The median age was 50 years (range, 14–85 years). Histologically, 98.9% of patients had nonkeratinizing NPC, 0.5% had keratinizing NPC, and the remainder (0.5%) had other types. All patients underwent pretreatment evaluation that included complete history, physical and neurological examinations, hematology and biochemistry profiles, nasopharyngeal fiberscopy, nasopharynx and neck MRI, chest radiography, and abdominal sonography. Medical and imaging records were retrospectively reviewed and all patients were restaged according to the 7^th^ edition of the AJCC staging system [5]. The TNM stage distribution of all patients was T1 in 19.7%, T2 in 10.1%, T3 in 37.8%, and T4 in 32.4%; N0 in 6.9%, N1 in 50.0%, N2 in 42.6%, and N3 in 0.5%; Stage I in 2.7%, Stage II in 14.9%, Stage III in 49.5%, and Stage IVA-B in 33.0%.

### Imaging

All patients underwent MRI using a 1.0 Tesla scanner (Siemens Magnetom Impact, Siemens Healthcare, Erlangen, Germany). The area from the suprasellar cistern to the inferior margin of the sternal end of the clavicle was examined using a head and neck segregate coil. T1-weighted fast spin-echo images in the axial, coronal, and sagittal planes (repetition time, 500 ms; echo time, 12 ms) and T2-weighted fast spin-echo MR images in the axial plane (repetition time, 3,304 ms; echo time, 96 ms) were obtained before the injection of contrast material. After intravenous injection of gadopentetate dimeglumine (Gd-DTPA; 0.1 mmol/kg body weight; Magnevist, Schering, Berlin, Germany), spin-echo T1-weighted axial and sagittal sequences and spin-echo T1-weighted fat-suppressed coronal sequences were performed sequentially using similar parameters to those used prior to Gd-DTPA injection. We used a section thickness of 5 mm and a 256 × 256 matrix size.

### Image assessment

Two radiologists with clinical focus on head and neck cancer and certifications for professional diagnostic imaging in China, who have been on staff for 10 years, evaluated the MR images separately. Any discord in evaluation was resolved by consensus every two weeks. Tumor soft tissue had low signal intensity on T1-weighted images; it had intermediate signal intensity superior to the muscle signal on T2-weighted images; tumor soft tissues had moderate enhancement on contrast-enhanced T1-weighted images, replacing the normal anatomy of the structure. Tumor regression was evaluated by the change in the maximum primary tumor diameter (MPTD) on the same plane after RT. The method applied to measure MPTD was as follows [9]: firstly, MPTD was measured on post-Gd-DTPA T1-weighted images. Secondly, tumor signal was not interrupted, but continuous on the maximum diameter. Finally, the maximum diameters in the axial, coronal and sagittal planes were measured separately; the largest value was recorded as the MPTD. Tumor regression was divided into four levels: CR, PR, SD, and PD, according to RECIST [13].

### Treatment

All patients were treated with definitive-intent RT. One hundred and five patients (55.9%) were treated with conventional 2-dimensional RT (2-DRT) and 83 (44.1%) were treated with 3-dimensional conformal RT (3-DCRT). The accumulated doses were 68–70 Gy to the gross tumor, 60–62 Gy to involved areas of the neck, and 50 Gy to uninvolved areas. Boost irradiation to the parapharyngeal space, skull-base, and primary or nodal sites were administered if indicated, and did not exceed 16 Gy. Ninety patients (57.7%) were treated with boost irradiation.

Platinum-based concomitant chemotherapy was administered to 130 patients with Stage III or Stage IVA-B disease (classified as T3-T4 or N2-N3). The remaining 25 patients with advanced-stage disease did not receive chemotherapy due to advanced age, heart disease, hepatitis, severe diabetes, inadequate renal function, patient refusal, or economic problems. When possible, salvage treatment (including afterloading, surgery, and chemotherapy) was provided in the event of documented relapse or if the disease persisted despite therapy.

### Statistical analysis

Patients were assessed every two months during the first year, every three months for the next two years, and every six months thereafter until death. All events were measured from the date of commencement of treatment. The following end points (time to the first defining event) were assessed: OS, local relapse-free survival (LRFS), distant metastasis-free survival (DMFS), and failure-free survival (FFS). Local recurrence was established by fiberoptic endoscopy and biopsy and/or MRI. Distant metastases were diagnosed based on clinical symptoms, physical examination, and imaging methods, including chest radiography, bone scan, computed tomography, and abdominal sonography.

All statistical analyses were performed using Statistical Package for the Social Sciences version 12.0 (SPSS, Chicago, IL, USA). Actuarial rates were calculated using the Kaplan-Meier method and differences were compared using the log-rank test. Multivariate analyses with the Cox proportional hazards model were used to test for independent significance by backward elimination of insignificant explanatory variables. Demographic characteristics (age, sex) were introduced into the models as covariates for all statistical tests. No correction for multiple testing was performed. The criterion for statistical significance was set at α = 0.05; *P*-values were based on two-sided tests.

## Results

### PT regression speed after RT and survival

**Figure 1:**
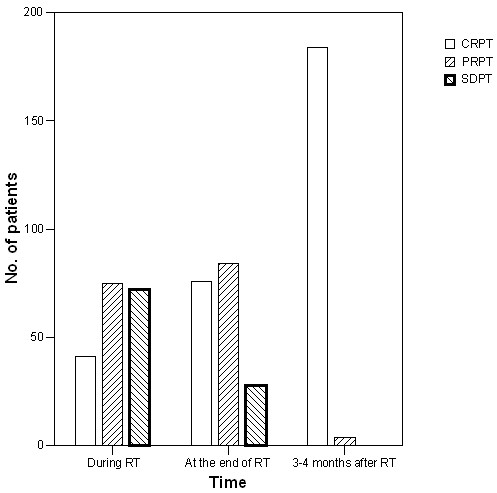
**Tumor regression of primary tumor after radiotherapy in 188 nasopharyngeal carcinoma patients.** CRPT, Complete response of primary tumor; PRPT, partial response of primary tumor; SDPT, stable disease of primary tumor.

Among 188 patients, 21.8% had CRPT, 39.9% had PRPT, and 38.3% had SDPT during RT. At the end of RT, 40.4% of patients had CRPT, 44.7% had PRPT, and 14.9% had SDPT. After 3–4 months of RT, 97.4% of patients had CRPT and 2.6% had persistent PT (Figure 1).

The median follow-up period for the whole group was 57 months (range, 2–110 months). Altogether, 41 patients (21.8%) had locoregional failure or distant metastases; 53 patients (28.2%) died. For all patients, the 5-year OS, FFS, DMFS, and LRFS rates were 72.1%, 76.8%, 85.1%, and 89.8%, respectively.

### Prognostic value of PT regression speed at the end of RT

**Figure 2:**
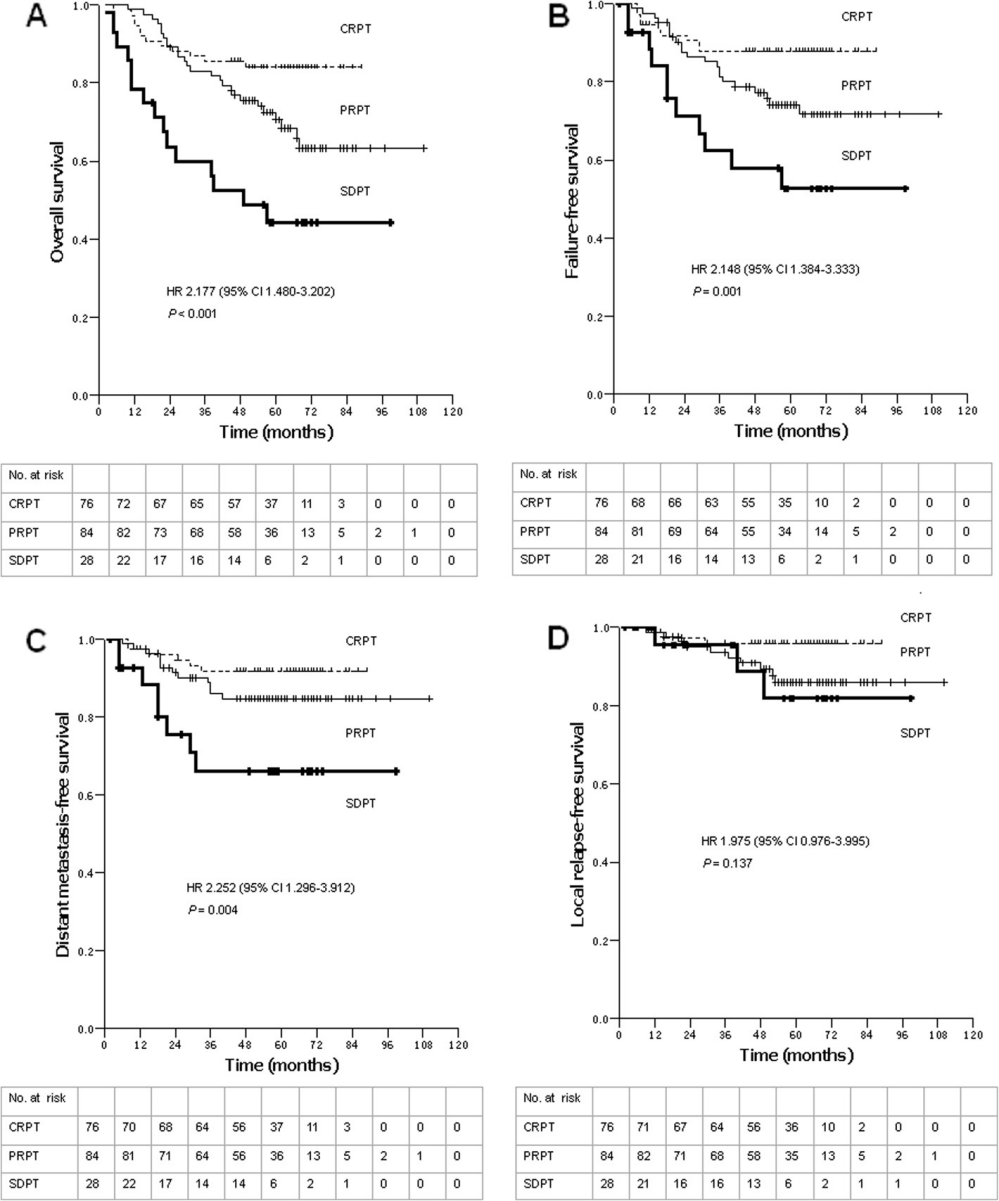
**Survival rates of 188 nasopharyngeal carcinoma patients. (A)** Overall survival, **(B)** failure-free survival, **(C)** distant metastasis-free survival, and **(D)** local relapse–free survival rates of patients with complete response of primary tumor (CRPT), partial response of primary tumor (PRPT), and stable disease of primary tumor (SDPT) at the end of radiotherapy. Hazard ratios (HR) were calculated with the unadjusted Cox proportional hazards model; *P*-values were calculated with the unadjusted log-rank test. 95% CI, 95% confidence interval.

Based on PT regression speed at the end of RT, the 188 patients were divided into three groups: CRPT, PRPT and SDPT. The 5-year OS rates for patients with CRPT, PRPT and SDPT were 84.0%, 70.7%, and 44.3%, respectively. The differences among these rates were highly significant (hazard ratio [HR] = 2.177, 95% confidence interval [CI] = 1.480-3.202; *P* < 0.001; Figure 2A). The 5-year FFS rates also differed significantly (87.8% vs. 74.3% vs. 52.7%, *P* = 0.001, HR = 2.148, 95% CI, 1.384-3.333, Figure 2B). Similarly, the 5-year DMFS rates were 91.7%, 84.7%, and 66.1%, respectively (HR = 2.252, 95% CI = 1.296-3.912; *P* = 0.004; Figure 2C). The 5-year LRFS rates were not significantly different (95.8% vs. 86.0% vs. 81.8%, *P =* 0.137, HR = 1.975, 95% CI, 0.976-3.995, Figure 2D).

To adjust for prognostic factors, the following parameters were introduced into the Cox regression model: age (≤ 50 vs. > 50 years), sex, chemotherapy (yes vs. no), radiation technique (2-DRT vs. 3-DCRT), boosting (yes vs. no), T stage (T1-2 vs. T3-4), N stage (N0-1 vs. N2-3), PT volume (< 19 cm^3^ vs. ≥ 19 cm^3^) and PT regression speed at the end of RT (CRPT vs. PRPT vs. SDPT). PT regression speed at the end of RT was the only independent prognostic factor of OS, FFS, and DMFS (*P <* 0.001, *P =* 0.001 and *P =* 0.004; Table 1).

**Table 1:** Multivariate analyses of prognostic factors in 188 NPC patients

**Endpoint**	**Variable**	**Estimate**	**HR**^**†**^	95% CI*	***P *** **value**^**‡**^
OS	Regression speed	0.778	2.177	1.480-3.203	< 0.001
FFS	Regression speed	0.764	2.148	1.384-3.333	0.001
DMFS	Regression speed	0.812	2.252	1.296-3.912	0.004
LRFS	Regression speed	0.681	1.975	0.976-3.995	0.058

^†^HR, Hazard ratio from Cox proportional hazards model; *CI, confidence interval; ^**‡**^*P*-values were calculated using an adjusted Cox proportional hazards model. OS, Overall survival; FFS, Failure-free survival; DMFS, Distant metastasis-free survival; LRFS, Local relapse-free survival. The following parameters were included in the model as covariates for each analysis: age (≤ 50 vs. > 50 years), sex, chemotherapy (yes vs. no), radiation technique (2-DRT vs. 3-DCRT), boosting (yes vs. no), T stage (T1-2 vs. T3-4), N stage (N0-1 vs. N2-3), primary tumor volume (< 19 cm^3^ vs. ≥ 19 cm^3^) and primary tumor regression speed at the end of RT (CRPT vs. PRPT vs. SDPT). Table contains only the results for primary tumor regression speed and other statistically significant variables.

### Prognosis comparison of patients with CRPT during RT and patients with CRPT only at the end of RT

Seventy-six patients with CRPT at the end of RT were divided into two groups: Group A (with CRPT during RT), 41 patients; Group B (with CRPT only at the end of RT), 35 patients. The 5-year OS, FFS, DMFS, and LRFS rates of Group A and B were 79.9% vs. 88.6% (*P =* 0.383), 80.2% vs. 97.1% (*P =* 0.033), 87.1% vs. 97.1% (*P =* 0.146), and 92.4% vs. 100% (*P =* 0.113), respectively, the details for which are listed in Table 2.

**Table 2:** Survival outcomes in patients with CRPT during RT and only at the end of RT

**Variable**	**CRPT during RT (N = 41)**	**CRPT at the end of RT (N = 35)**	**HR **^**† **^(95% CI*)	***P***^**‡**^
5-yr OS rate	79.9%	88.6%	0.590 (0.177-1.962)	0.383
5-yr FFS rate	80.2%	97.1%	0.144 (0.018-1.151)	0.033
5-yr DMFS rate	87.1%	97.1%	0.233 (0.027-1.992)	0.146
5-yr LRFS rate	92.4%	100%	0.018 (0.000-206.004)	0.113

^†^HR, Hazard ratios calculated using the unadjusted Cox proportional hazards model; *CI, confidence interval; ^‡^*P*-values were calculated by the unadjusted log-rank test. RT*,* Radiotherapy; OS*,* Overall survival; FFS*,* Failure-free survival; DMFS*,* Distant metastasis-free survival; LRFS*,* Local relapse-free survival; CRPT*,* Complete response of primary tumor.

### Value of boost irradiation in patients with persistent PT at the end of RT

One hundred and twelve patients had persistent PT at the end of RT; 27 were not treated with boost irradiation (Group C) and 85 were treated with boost irradiation (Group D). The 5-year OS, FFS, DMFS, and LRFS rates of Group C and D were 65.5% vs. 63.9% (*P =* 0.477), 73.1% vs. 67.7% (*P =* 0.579), 84.4% vs. 79.2% (*P =* 0.589), and 87.1% vs. 84.6% (*P =* 0.812), respectively; the details are listed in Table 3.

**Table 3:** Prognosis of patients with persistent PT at RT end with and without boost irradiation

**Variable**	**Without boost irradiation (N = 27)**	**With boost irradiation (N = 85)**	**HR **^**† **^(95% CI*)	***P***^**‡**^
5-yr OS rate	65.5%	63.9%	1.308 (0.622-2.748)	0.477
5-yr FFS rate	73.1%	67.7%	1.267 (0.547-2.934)	0.579
5-yr DMFS rate	84.4%	79.2%	1.350 (0.451-4.039)	0.589
5-yr LRFS rate	87.1%	84.6%	1.170 (0.322-4.252)	0.812

^†^HR, Hazard ratios calculated using the unadjusted Cox proportional hazards model; *CI, confidence interval; ^‡^*P*-values were calculated by the unadjusted log-rank test. PT*,* Primary tumor; RT*,* Radiotherapy; OS*,* Overall survival; FFS*,* Failure-free survival; DMFS*,* Distant metastasis-free survival; LRFS*,* Local relapse-free survival.

## Discussion

In NPC patients, the determination of a treatment regimen is mainly based on tumor stage. Clinical response is usually evaluated 3–4 months after RT. The lack of monitoring for tumor response during RT results in patients with poor tumor regression not receiving immediate strengthening treatment.

### PT regression speed after RT

There are two treatment response evaluation criteria for solid tumors: that of the World Health Organization (WHO) and RECIST [13]. The WHO criteria adopt bidimensional measurement; RECIST adopts unidimensional measurement. Compared to the WHO criteria, that of RECIST underscores reevaluation and follow-up. Therefore, we adopted the RECIST criteria. In this study, 97.4% of patients had CRPT after 3–4 months of RT, similar to the 96.5%–100% reported in previous studies [14-16]. Further, we considered it important to observe the speed of CRPT. Although only 21.8% of patients had CRPT during RT and 40.4% of patients had CRPT at the end of RT, 97.4% of patients had CRPT after 3–4 months of RT. In the study of Kwong et al., 803 patients underwent serial post-RT nasopharyngeal biopsies. A high proportion of early positive histology remitted spontaneously, and 93.1% of patients had negative histology at week 12 after RT [17]. The phenomenon is well known as delayed regression.

### Prognostic value of PT regression speed at the end of RT

In this study, PT regression speed at the end of RT was an independent prognostic factor of OS, FFS, and DMFS. In addition, CRPT at the end of RT suggested the best prognosis, followed by PRPT, and SDPT suggested the worst prognosis. Mäntylä et al. reported that, in head and neck cancers, prognosis was significantly more favorable when an advanced tumor had disappeared at the end of treatment [18]. Strengthening treatment may be administered immediately to patients with poor tumor regression. Although CCRT is the standard treatment for locoregionally advanced NPC, the role of adjuvant chemotherapy is debated [19-26]. The Phase III multicentre randomized controlled trial by Chen et al. helped resolve this issue, demonstrating that adjuvant chemotherapy did not contribute any benefit to CCRT. However, the median follow-up was only 37.8 months, and longer follow-up is needed to fully assess survival and late toxic effects [27]. Adjuvant chemotherapy, an important chemotherapy regimen, should not be abandoned in NPC patients; instead, it may be used to assess whether it confers survival benefit to patients with obvious persistent tumor at the end of RT in clinical trials.

The 5-year LRFS rates among the CRPT, PRPT, and SDPT groups were not significantly different (95.8% vs. 86.0% vs. 81.8%, *P =* 0.137). The study by Jaulerry et al. demonstrated that tumor regression during external RT was an independent predictive factor of local control in head and neck carcinomas [28]. Bataini et al. also confirmed that complete tumor clearance following RT is a reliable indicator of permanent local control for squamous cell carcinoma of the oropharynx and pharyngolarynx [29]. The differing results might be due to the following reasons: first, NPC possesses obviously unique pathological types as compared to other head and neck cancers, where > 95% of patients in endemic areas have nonkeratinizing NPC [30]. The treatment method and prognosis for this histologic subtype is very different from that of other subtypes. Second, distant metastasis, which is the main reason for treatment failure in NPC patients, usually occurs in the first two years after treatment [31]. Such patients might die due to distant metastasis before possible local relapse. Third, 5-year LRFS rates are as high as 86.8-94.9% in NPC patients [1,4]. This justifies the call for enrollment of a large number of patients for observing significant statistical differences in LRFS rates among groups. The 5-year LRFS rates for patients with CRPT, PRPT, and SDPT gradually declined (95.8% vs. 86.0% vs. 81.8%, *P =* 0.137), but with no statistical difference. However, the number of patients in the three groups was small, which might have led to false-negative results.

### Prognostic comparison of rapid and slow regression

We compared the prognosis of patients with CRPT when the radiation dose was 46–50 Gy and those with CRPT only at the end of RT. The OS, DMFS, and LRFS rates of patients in the latter group were higher than that of patients in the former group, but the *P*-values were not significantly different; however, the FFS rate was significantly different (80.2% vs. 97.1%, *P =* 0.033). Wang et al. confirmed that the tumor regression rate during radical RT had significant predictive value for 5-year OS, FFS, DMFS and locoregional FFS. In that study, the prognosis of slow response was significantly better than that of rapid regression [32]. The exact radiobiological mechanism through which tumor regression speed influences prognosis in NPC patients remains unknown. Patients with rapid tumor regression might have higher incidence rates of distant metastasis. Li et al. divided NPC into four RT-related types: radiosensitive and non–metastasis-prone, radioresistant and non–metastasis-prone, radiosensitive and metastasis-prone, and radioresistant and metastasis-prone [33]. Local control was better when a tumor was radiosensitive and poorer when a tumor was radioresistant. As distant metastasis could also result in treatment failure, a curative effect might not be achieved in radiosensitive tumors for which excellent local control is obtained [33].

### Value of boost irradiation in patients with persistent PT at the end of RT

In 1999, Tate et al. reported that stereotactic radiosurgical boost following fractionated external beam RT (EBRT) provided excellent local control in advanced-stage NPC [34]. Subsequent studies also supported the premise that stereotactic RT boost after EBRT results in excellent local control for patients with NPC [35,36]. Leung et al. and Yeo et al. both reported that brachytherapy boost supplementing radical EBRT improved local control in NPC patients with early T1-2B disease [37,38]. However, Schinagl et al. reported that while EBRT with endocavitary brachytherapy produced excellent rates of local control for T1-2 tumors, the high incidence of late toxicity suggested overtreatment [39]. In this study, boost irradiation did not confer any survival benefit to patients with persistent PT at the end of RT. It has been recommended that boost radiation to residual PTs be withheld unless positive biopsy samples persist at ≥10 weeks after RT [40].

### Limitations

Due to limited medical resources, 2-DRT and 3-DCRT were used instead of IMRT. Although excellent local control can be achieved in NPC by IMRT, distant metastasis remains the major cause of treatment failure [1,2,4]. The number of patients in the two-subgroup analysis of prognostic comparison of patients with CRPT during RT to patients with CRPT only at the end of RT and the value of boost irradiation in patients with persistent PT at the end of RT was too small, which might have led to false-negative results. Moreover, this was a retrospective study, and the conclusions need to be confirmed by future prospective studies.

## Conclusions

PT regression speed at the end of RT was an independent prognostic factor of OS, FFS, and DMFS in NPC patients. Immediate strengthening treatment may be administered to patients with poor tumor regression at the end of RT. Patients with fast tumor regression could obtain excellent local control but a curative effect might still not be achieved due to distant metastasis. There is a need for awareness of the requirement of boost irradiation after radical RT because of the high incidence of late toxicity. However, this was a retrospective study with a limited number of patients, and future studies focusing on tumor regression speed and its prognostic impact are warranted.

## Competing interests

The authors declare that they have no competing interests.

## Authors’ contributions

NZ and S-BL participated in literature research, study design, data collection, data analysis, interpretation of findings and the draft of the manuscript. Y-MD, H-YC, Z-QL, SQL and LY carried out the data collection. R-LL and HZ reviewed MR images. D-SL performed the statistical analysis. YC contributed with study design, data collection, interpretation of findings and critical edit of the manuscript. All authors read and approved the final manuscript.

## Pre-publication history

The pre-publication history for this paper can be accessed here:

http://www.biomedcentral.com/1471-2407/14/136/prepub
